# Metabolomic Elucidation of the Effects of Curcumin on Fibroblast-Like Synoviocytes in Rheumatoid Arthritis

**DOI:** 10.1371/journal.pone.0145539

**Published:** 2015-12-30

**Authors:** Joong Kyong Ahn, Sooah Kim, Jiwon Hwang, Jungyeon Kim, You Sun Lee, Eun-Mi Koh, Kyoung Heon Kim, Hoon-Suk Cha

**Affiliations:** 1 Department of Internal Medicine, Division of Rheumatology, Kangbuk Samsung hospital, Sungkyunkwan University School of Medicine, Seoul, Republic of Korea; 2 Department of Biotechnology, Graduate School, Korea University, Seoul, Republic of Korea; 3 Department of Internal Medicine, National Police Hospital, Seoul, Republic of Korea; 4 Department of Internal Medicine, Division of Rheumatology, Samsung Changwon Hospital, Sungkyunkwan University School of Medicine, Changwon, Republic of Korea; 5 Department of Medicine, Division of Rheumatology, Samsung Medical Center, Sungkyunkwan University School of Medicine, Seoul, Republic of Korea; University of Texas Health Science Center at Houston, UNITED STATES

## Abstract

Rheumatoid arthritis (RA) is a chronic systemic inflammatory disease characterized by synovial inflammation and joint disability. Curcumin is known to be effective in ameliorating joint inflammation in RA. To obtain new insights into the effect of curcumin on primary fibroblast-like synoviocytes (FLS, N = 3), which are key effector cells in RA, we employed gas chromatography/time-of-flight mass spectrometry (GC/TOF-MS)-based metabolomics. Metabolomic profiling of tumor necrosis factor (TNF)-α-stimulated and curcumin-treated FLS was performed using GC/TOF-MS in conjunction with univariate and multivariate statistical analyses. A total of 119 metabolites were identified. Metabolomic analysis revealed that metabolite profiles were clearly distinct between TNF-α-stimulated vs. the control group (not stimulated by TNF-α or curcumin). Treatment of FLS with curcumin showed that the metabolic perturbation by TNF-α could be reversed to that of the control group to a considerable extent. Curcumin-treated FLS had higher restoration of amino acid and fatty acid metabolism, as indicated by the prominent metabolic restoration of intermediates of amino acid and fatty acid metabolism, compared with that observed in TNF-α-stimulated FLS. In particular, the abundance of glycine, citrulline, arachidonic acid, and saturated fatty acids in TNF-α-stimulated FLS was restored to the control level after treatment with curcumin, suggesting that the effect of curcumin on preventing joint inflammation may be elucidated with the levels of these metabolites. Our results suggest that GC/TOF-MS-based metabolomic investigation using FLS has the potential for discovering the mechanism of action of curcumin and new targets for therapeutic drugs in RA.

## Introduction

Rheumatoid arthritis (RA) is a chronic systemic inflammatory disease characterized by synovial inflammation and hyperplasia, and concomitant destruction of the cartilage and bone. Proinflammatory transcription factors such as NF-κB and proinflammatory cytokines such as tumor necrosis factor (TNF)-α, are closely associated with the pathological process of RA [1].

Curcumin (*diferuloylmethane*), a polyphenol derived from the rhizomes of the plant *Curcuma longa* (turmeric), has been used as a traditional medicine to treat many inflammatory disorders [2,3]. Many researchers have shown the potent anti-inflammatory, anti-carcinogenic, and antioxidant action of curcumin against cancer and inflammatory diseases [3–7]. Human clinical trials have also shown beneficial effects against cancer and inflammatory diseases, such as inflammatory bowel disease, uveitis, and orbital pseudotumor [3,8]. Despite the beneficial effects of curcumin in cancer and inflammatory diseases, it has not yet been approved to treat chronic inflammatory arthritis such as RA.

Curcumin is also effective in reducing joint inflammation, based on studies conducted in fibroblast-like synoviocytes (FLS) and animal models in RA [9–12]. Although the exact mechanism underlying the effect in inflammatory diseases remains to be elucidated, the anti-inflammatory activity of curcumin appears to be closely related to the suppression of proinflammatory cytokines such as TNF-α, interleukin (IL)-1β and the down-regulation of cyclooxygenases (COX)-2, nitric oxide synthase, mitogen-activated kinases, and NF-κB [3,8,13,14].

In this study, we applied metabolomics to investigate the beneficial effect of curcumin on FLS in RA. Metabolomics is a tool for comprehensively analyzing all small-molecule metabolites generated in a given biological system, and has been widely used in several biomedical areas, such as cellular responses to drugs or nutrients and new drug development [15]. For example, metabolomics has provided insight into the mechanism of action underlying curcumin in breast cancer cell lines [16,17].

The important pathologic feature of RA FLS is the characteristic ability to express inflammatory cytokines, chemokines, adhesion molecules, and matrix-degrading enzymes. FLS also increase in number and become a prominent component of the destructive pannus in RA [18,19]. Although the clinical activity of curcumin is well known, the mechanism of action of curcumin remains to be elucidated at the cellular level [16,20]. In this study, through metabolomic analysis using FLS with the same genetic background and treated in a uniform manner, a deep and new understanding of the therapeutic effects of curcumin in RA was targeted.

## Materials and Methods

### Preparation of Curcumin

Curcumin was purchased from Sigma-Aldrich (St. Louis, MO, USA). Curcumin 10mg was dissolved in dimethyl sulfoxide (DMSO) 1mL, and then further diluted in phosphate-buffered saline (PBS).

### Isolation and culture of RA FLS

Because immortalized mammalian FLS cell lines in RA are not yet available for scientific research, primary FLS cultures were employed. Synovial tissues were obtained from 3 RA patients undergoing arthroscopic wrist synovectomy or total knee joint replacement. The clinical characteristics of the patients are depicted in Table A in S1 File. The experimental protocols used in this study were approved by the Samsung Medical Center (#2006-08-117) institutional review board and written informed consent was obtained from each patient included in this study. This study was conducted in accordance with the principles expressed in the Helsinki Declaration.

Patients with RA were diagnosed according to the standard criteria reported by the American College of Rheumatology [21]. Synovial tissue was minced, and digested overnight with 5 mg/mL type IV collagenase (Sigma, Poole, UK) and 150 μg/mL type I DNase (Sigma, Poole, UK), and separated from undigested tissue using unit gravity sedimentation. After collecting the suspended cells into fresh tubes, the cells were harvested by centrifugation at 500 *g* (relative centrifuge force) for 10 min. The pellet was washed twice with Dulbecco’s Modified Eagle’s Media (DMEM; Life Technologies, Grand Island, NY, USA) containing 10% fetal bovine serum (FBS). The resuspended cells were plated at a concentration of 2 x 10^6^/mL in a total volume of 1 mL/200 mm^2^ into T-25 culture flasks. After overnight incubation, the non-adherent cells were removed by replacing the medium with fresh culture medium. The attached cells were cultured in DMEM with 10% FBS and 50 units/mL penicillin, 50 mg/mL streptomycin, and 0.025 mg/mL amphotericin B, until 90% confluent growth was achieved. Primary cultured cells were passaged three to five times over several weeks for subsequent experiments. Between the third and fourth passages, cultures were a homogeneous population of fibroblastic cells.

To investigate the metabolic perturbation and anti-inflammatory effects of curcumin on RA FLS, FLS were divided into three groups as follows: 1) RA FLS pretreated with curcumin (40 μM) for 1hr before stimulation with TNF-α (100 ng/ml) for 24 hour; 2) RA FLS only stimulated with TNF-α (100 ng/ml) for 24 hours; 3) control RA FLS not treated with curcumin or TNF-α at all.

### Treatment of RA FLS with curcumin and TNF-α

To study the effects of curcumin on IL-6, IL-8, and matrix metalloproteinase (MMP) production, RA FLS (N = 3) were incubated with DMSO-containing vehicle or curcumin (40 μM) for 1 h, followed by stimulation with TNF-α (100 ng/mL) for 24 h. Then, the culture supernatant was collected and centrifuged at 10,000 *g* for 5 min at 4°C to remove particulate matter, and was stored at -20°C in fresh tubes. Using commercially available enzyme immunoassay kits (ELISA; R&D Systems, Minneapolis, MN, USA), the culture supernatants were used to determine the quantities of IL-6, IL-8, MMP-1, and MMP-3.

### Sample preparation for metabolomics

To obtain the metabolome sample from RA FLS (N = 3), we used fast filtration methods with a slight modification of the methods used in previous studies [22]. Briefly, RA FLS were detached in PBS buffer using a rubber-tipped cell scraper. The 2 mL of FLS were vacuum-filtered using a nylon membrane filter (0.45-μm pore size, 30 mm diameter; Whatman, Piscataway, NJ, USA) and washed with 2 mL PBS buffer. The cells with membrane filter were mixed with a water:2-propanol:methanol mixture (5:2:2, v/v/v) at -20°C, and then, the extraction mixture was directly immersed in liquid nitrogen. The extraction mixture was thawed on ice and vortexed for 3 min. After centrifugation at 13,000 *g* for 5 min at 4°C, the supernatant was collected and completely vacuum-dried using a vacuum concentrator (Labconco, Kansas City, MO, USA). The dried samples were derivatized with 5 μL of methoxyamine hydrochloride in pyridine (40 mg/mL; Sigma-Aldrich, St. Louis, MO, USA) at 30°C for 90 min and 45 μL of N-methyl-N-(trimethylsilyl) trifluoroacetamide (Fluka; Buchs, Switzerland) at 37°C for 30 min. A mixture of fatty acid methyl esters (C08-C30) as retention index markers was added to the derivatized extract.

### Metabolite analysis using gas chromatography with time-of-flight mass spectrometry (GC/TOF-MS)

We used an Agilent 7890B GC (Agilent Technologies, Wilmington, DE, USA) equipped with Pegasus HT TOF-MS (LECO, St. Joseph, MI, USA) for sample analysis. A 1 μL sample was injected into an RTX-5Sil MS column (30 m × 0.25 mm, 0.25-μm film thickness; Restek, Bellefonte, PA, USA) and an integrated guard column (10 m × 0.25 mm, 0.25-μm film thickness; Restek, Bellefonte, PA, USA) on splitless mode for separation of metabolome in samples. The oven temperature was set for 5 min at 50°C, and was then increased to 330°C at the rate of 20°C/min, where it was then held for 5 min. The mass spectra were collected at the mass range of 85 to 500 *m/z* at an acquisition rate of 17 spectra/s. The temperatures of ion source and transfer line were 250°C and 280°C, respectively, and the impact of electron ionization was at 70 eV.

### Statistical analysis

The data obtained from GC/TOF-MS was preprocessed by using Chroma TOF software (v. 4.50; LECO, St. Joseph, MI, USA). After that, we used BinBase, an in-house database for further processing of the preprocessed data [23]. The processed data were then normalized by abundance of the sum of identified metabolites. The median of coefficient of variation of the identified metabolites in control group, TNF-α-stimulated, and curcumin-treated RA FLS were 0.21, 0.10, and 0.09, respectively. Therefore, all identified metabolites were used for statistical analyses in this study. We conducted univariate and multivariate statistical analyses of normalized data using Statistica (v. 7.1; StatSoft, Tulsa, OK, USA) [24]. After transforming the data with unit variance scaling, hierarchical clustering analysis (HCA) based on the Euclidean distance coefficient and average linkage method was performed using MultiExperiment Viewer (MeV; Dana-Farber Cancer Institute, Boston, MA, USA) to cluster both conditions and the identified metabolites [25]. The pathway analysis was conducted using the web-based MetaboAnalyst (http://www.metaboanalyst.ca) [26]. The measured levels of IL-6, IL-8, and MMP were expressed as mean ± standard deviation for the number of experiments. Statistical significance was determined by comparisons among each group using the Mann-Whitney test or the Kruskal-Wallis test. Values with a *P* < 0.05 were considered significant.

## Results

### Overview of metabolite profiles among untreated, TNF-α-stimulated, and curcumin-treated RA FLS

The metabolites obtained from the control group, TNF-α-stimulated, and curcumin-treated RA FLS were analyzed using GC/TOF–MS, and over 1,000 unique *m/z* values with retention time were detected. After deconvolution and alignment using ChromaTOF software and BinBase, a total of 119 metabolites were identified (Table 1). These metabolites are classified into various chemical classes, including amino acids (19.3% of identified metabolites), organic acids (19.3%), sugars and sugar alcohols (18.5%), fatty acids (17.6%), and amines (12.6%). These metabolites are major intermediates of metabolic pathways, such as glycolysis, amino acid and fatty acid metabolisms, as well as those of the urea and tricarboxylic acid cycles.

**Table 1:** Classification of 119 metabolites identified according to metabolic pathway and comparison of relative levels of metabolites between three groups.

Metabolite	Control	TNF-α-stimulated	Curcumin + TNF-α-stimulated
**Amine**			
5ʹ-deoxy-5ʹ-methylthioadenosine	1 ± 0.228	0.345 ± 0.042*	0.973 ± 0.340
adenosine	1 ± 0.283	0.433 ± 0.053#	1.227 ± 0.239*
guanine	1 ± 0.059	0.492 ± 0.165*	0.909 ± 0.107#
inosine	1 ± 0.499	0.516 ± 0.316	1.004 ± 0.097
methylamine NIST^a^	1 ± 0.513	0.333 ± 0.102	0.683 ± 0.162#
O-phosphorylethanolamine	1 ± 0.172	0.736 ± 0.029	1.171 ± 0.210#
spermidine	1 ± 0.319*	0.788 ± 0.559	2.321 ± 0.165#
thymine	1 ± 0.367	0.662 ± 0.493	0.579 ± 0.155
uracil	1 ± 0.239	0.999 ± 0.107	0.814 ± 0.091
xanthine	1 ± 0.013	0.745 ± 0.037*	0.682 ± 0.234
citrulline	1 ± 0.384	3.365 ± 0.263*	0.883 ± 0.085*
cysteine	1 ± 0.200*	1.020 ± 0.210	2.333 ± 0.082*
hypoxanthine	1 ± 0.691	1.917 ± 0.167	1.260 ± 0.097*
nicotinamide	1 ± 0.111	1.513 ± 0.014#	1.076 ± 0.151#
putrescine	1 ± 0.219	1.644 ± 0.119#	0.996 ± 0.093*
**Amino acid**			
asparagine	1 ± 0.148	0.604 ± 0.317	0.978 ± 0.127
asparagine dehydrated	1 ± 0.275	0.597 ± 0.136	0.750 ± 0.717
aspartate	1 ± 0.400#	0.427 ± 0.044	2.715 ± 0.031*
β-alanine	1 ± 0.166	0.649 ± 0.141#	1.140 ± 0.178#
alanine	1 ± 0.312	1.185 ± 0.329	1.046 ± 0.218
glutamate	1 ± 0.054*	1.003 ± 0.024	2.381 ± 0.032*
glutamine	1 ± 0.177	1.630 ± 0.099*	1.183 ± 0.018#
glycine	1 ± 0.162	1.354 ± 0.003#	1.038 ± 0.051*
histidine	1 ± 0.440	15.36 ± 2.694*	3.308 ± 2.543*
isoleucine	1 ± 0.178	2.155 ± 0.040*	1.285 ± 0.016*
leucine	1 ± 0.258	2.420 ± 0.177*	1.526 ± 0.059*
lysine	1 ± 0.147	1.814 ± 0.101*	0.940 ± 0.027*
methionine	1 ± 0.262	1.617 ± 0.351	0.798 ± 0.169#
N-methylalanine	1 ± 0.206	1.212 ± 0.235	1.025 ± 0.269
ornithine	1 ± 0.309	1.928 ± 0.016#	1.034 ± 0.100*
oxoproline	1 ± 0.030	1.607 ± 0.005*	1.046 ± 0.011*
phenylalanine	1 ± 0.012*	1.612 ± 0.078*	0.942 ± 0.011*
proline	1 ± 0.391#	1.397 ± 0.140	1.997 ± 0.129*
serine	1 ± 0.044*	1.523 ± 0.018*	1.125 ± 0.010*
threonine	1 ± 0.031*	1.691 ± 0.038*	1.147 ± 0.018*
tryptophan	1 ± 0.097	1.862 ± 0.057*	1.049 ± 0.004*
tyrosine	1 ± 0.064	2.096 ± 0.032*	1.030 ± 0.017*
valine	1 ± 0.146#	2.201 ± 0.092*	1.349 ± 0.066*
**Fatty acid**			
1-monopalmitin	1 ± 0.340	0.193 ± 0.018#	1.206 ± 0.041*
2-ketoisocaproic acid	1 ± 0.439	0.841 ± 0.195	0.801 ± 0.156
arachidic acid	1 ± 0.181	0.457 ± 0.076*	0.847 ± 0.101*
arachidonic acid	1 ± 0.190#	0.347 ± 0.109*	0.644 ± 0.057#
behenic acid	1 ± 0.744	0.525 ± 0.051	0.386 ± 0.414
capric acid	1 ± 0.083*	0.783 ± 0.386	1.277 ± 0.043
fructose-6-phosphate	1 ± 0.211	0.483 ±0.083#	1.984 ±0.826#
heptadecanoic acid	1 ± 0.258	0.466 ± 0.028	1.052 ± 0.088*
lauric acid	1 ± 0.176	0.614 ± 0.027	0.760 ± 0.013*
lignoceric acid	1 ± 0.232#	0.478 ± 0.103#	0.605 ± 0.033
linoleic acid	1 ± 0.273	0.629 ± 0.054	0.488 ± 0.039#
linolenic acid	1 ± 0.402	0.476 ± 0.059	0.746 ± 0.067*
myristic acid	1 ± 0.141	0.657 ± 0.011	1.058 ± 0.022*
octadecanol	1 ± 0.035	0.523 ± 0.116*	1.153 ± 0.152*
oleic acid	1 ± 0.224	0.410 ± 0.037#	1.023 ± 0.119*
palmitic acid	1 ± 0.076	0.534 ± 0.045*	0.949 ± 0.113*
palmitoleic acid	1 ± 0.212	0.531 ± 0.302	0.839 ± 0.190
pelargonic acid	1 ± 0.150	0.595 ± 0.144#	1.121 ± 0.152#
pentadecanoic acid	1 ± 0.739	3.336 ± 0.242*	1.703 ± 0.753#
stearic acid	1 ± 0.085	0.533 ± 0.034*	0.922 ± 0.100*
**Organic acid**			
2-hydroxyvalerate	1 ± 0.252	0.577 ± 0.221	0.917 ± 0.112
2-ketoadipate	1 ± 0.456	0.322 ± 0.152	0.619 ± 0.148
3-phenyllactate	1 ± 0.178	0.357 ± 0.032*	0.693 ± 0.169#
adipate	1 ± 0.357	0.319 ± 0.055	0.870 ± 0.174*
benzoate	1 ± 0.380	0.404 ± 0.246	0.762 ± 0.010
β-hydroxybutyrate	1 ± 0.264	0.935 ± 0.169	1.177 ± 0.486
fumarate	1 ± 0.371*	0.962 ± 0.028	2.254 ± 0.132*
galactonate	1 ± 0.099#	0.951 ± 0.637	1.483 ± 0.214*
gluconate	1 ± 0.095*	0.238 ± 0.400	0.016 ± 0.001
glycerate	1 ± 0.067	0.815 ± 0.142	0.900 ± 0.068
indole-3-lactate	1 ± 0.068#	0.572 ± 0.052*	0.746 ± 0.111
lactate	1 ± 0.048	0.884 ± 0.052#	0.924 ± 0.023
malate	1 ± 0.282#	0.656 ± 0.102	1.511 ± 0.111*
malonate	1±0.027	0.473 ± 0.051*	1.135 ± 0.119*
oxalate	1 ± 0.211	0.512 ± 0.044	0.929 ± 0.007*
phenylacetate	1 ± 0.367	0.612 ± 0.196*	0.939 ± 0.081
phosphogluconate	1 ± 0.392	0.699 ± 0.122	1.815 ± 0.251*
pyrrole-2-carboxylate	1 ± 0.177	0.630 ± 0.110#	1.171 ± 0.154*
pyruvate	1 ± 0.181	0.496 ± 0.045*	0.639 ± 0.137
terephthalate	1 ± 0.343	0.894 ± 0.011	1.070 ± 0.044*
aminomalonate	1 ± 0.219#	1.159 ± 0.048	1.947 ± 0.012*
citramalate	1 ± 0.255	1.116 ± 0.044	0.801 ± 0.049*
succinate	1 ± 0.181	1.453 ± 0.002#	1.061 ± 0.009*
**Phosphate**			
adenosine-5-monophosphate	1 ± 0.287	0.256 ± 0.055#	1.052 ± 0.030*
β-glycerolphosphate	1 ± 0.342	0.970 ± 0.049	1.018 ± 0.363
mannose-6-phosphate NIST^a^	1 ± 0.272	0.621 ± 0.261	0.906 ± 0.355
phosphate	1 ± 0.015*	0.624 ± 0.014*	0.941 ± 0.009*
pyrophosphate	1 ± 0.344*	0.292 ± 0.022	3.546 ± 0.213*
glucose-6-phosphate	1 ± 0.127	1.290 ± 0.916	1.334 ± 0.228
**Sugar and sugar alcohols**			
3,6-anhydro-D-galactose	1 ± 0.065	0.327 ± 0.022*	0.944 ± 0.247#
cellobiose	1 ± 0.625	0.540 ± 0.121	1.419 ± 0.054*
citrate	1 ± 0.182*	0.940 ± 0.027	1.747 ± 0.029*
glycerol	1 ± 0.521	0.659 ± 0.108	1.235 ± 0.101*
glycolate	1 ± 0.073#	0.714 ± 0.147#	1.183 ± 0.017#
lactulose	1 ± 0.142#	0.579 ± 0.303	0.467 ± 0.190
maltotriose	1 ± 0.461	0.397 ± 0.072	0.828 ± 0.171#
mannose	1 ± 0.089	0.654 ± 0.007*	0.965 ± 0.020*
palatinitol	1 ± 0.286	0.443 ± 0.148#	0.958 ± 0.089*
phytol	1 ± 0.271	0.449 ± 0.051#	0.835 ± 0.071*
threose	1 ± 0.260	0.593 ± 0.050*	2.274 ± 0.142*
xylose	1 ± 0.098#	0.786 ± 0.846	1.448 ± 0.141
lyxose	1 ± 0.752	1.465 ± 1.221	0.893 ± 0.690
mannitol	1 ± 0.488	1.406 ± 0.087	0.475 ± 0.005*
melibiose	1 ± 0.361	1.105 ± 0.189	1.147 ± 0.240
myo-inositol	1 ± 0.126*	1.099 ± 0.018	1.839 ± 0.021*
arabitol	1 ± 0.237	1.067 ± 0.191	0.942 ± 0.073
fructose	1 ± 0.064#	1.740 ± 0.033*	1.137 ±0.040*
galactose	1 ± 0.123	1.892 ± 0.073*	1.063 ± 0.019
glucose	1 ± 0.113	1.672 ± 0.043*	1.036 ± 0.056*
tagatose	1 ± 0.126	1.835 ± 0.044*	1.127 ± 0.029*
threitol	1 ± 0.013	1.501 ± 0.011*	0.991 ± 0.017*
**Others**			
tyrosol	1 ± 0.091	0.462 ± 0.082*	1.080 ± 0.149*
benzamide	1 ± 0.177	0.413 ± 0.286#	0.942 ± 0.051
2,3-butanediol NIST^a^	1 ± 0.256	0.538 ± 0.076#	0.784 ± 0.069#
carnitine	1 ± 0.204	0.511 ± 0.491	1.104 ± 0.179
dihydroxyacetone	1 ± 0.322	0.317 ± 0.017#	0.886 ± 0.078*
salicylaldehyde	1 ± 0.455	0.329 ± 0.086	0.589 ± 0.023#
squalene	1 ± 0.028	0.405 ± 0.038*	0.834 ± 0.216#
taurine	1 ± 0.293	0.649 ± 0.106	1.899 ± 0.697#
urea	1 ± 0.243*	0.001 ± 0.000*	0.002 ± 0.000*

Mean control value is set to 1 while dispersion of control data is maintained. Unless otherwise indicated, values are mean ± standard deviation.

In comparison between Control vs. TNF-α or TNF-α vs. Curcumin pretreatment, or Curcumin pretreatment vs. Control, significant difference is observed in the TNF-α-stimulated, curcumin treatment, and control groups, respectively.

#, *P* < 0.05

*, *P* < 0.01

^a^The metabolite identified by National Institute of Standards and Technology (NIST) mass spectral library, but not verified by its authentic standard chemical.

### Principal component analysis (PCA) and hierarchical cluster analysis (HCA) of metabolomic data

**Fig 1:**
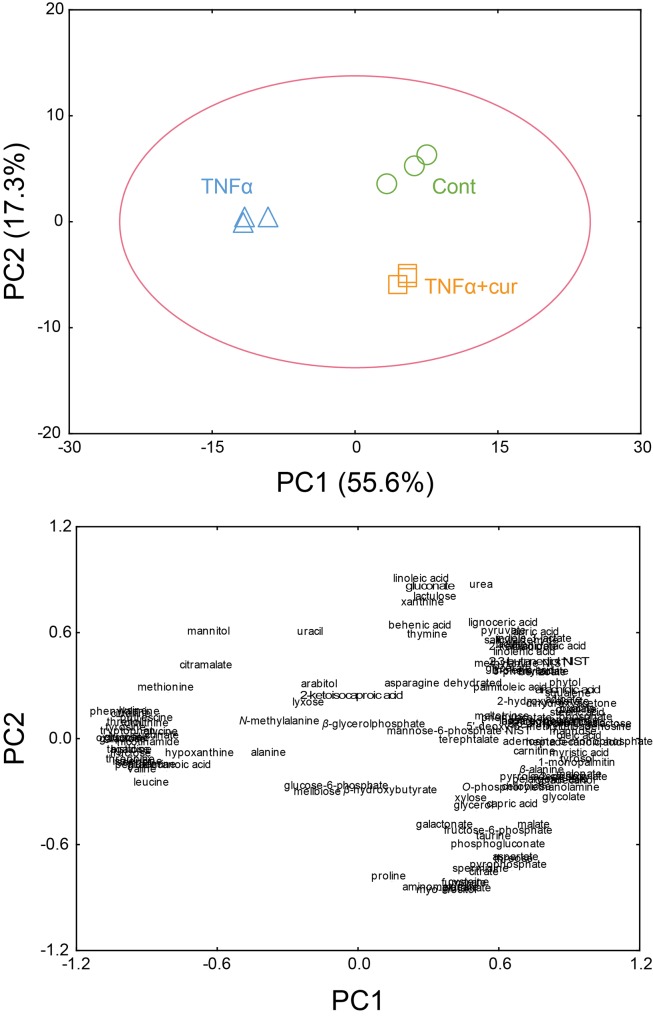
PCA score (A) and loading plots (B) of RA fibroblast-like synoviocytes (FLS), which were not stimulated (Control), stimulated with TNF-α (TNF), and treated with curcumin (Curcumin). (A) Principal component (PC)1 explained the significant separation of metabolite profiles between the TNF-α-stimulated group on the negative region of the PC1, and the control and curcumin-treated groups on the positive region of the PC1. Further, the control group was clearly separated from the curcumin-treated group on PC2. (B) PC1 was explained by 84 metabolites that correlated positively with the axis, and 35 metabolites that correlated negatively.

After the normalization by the sum of peak intensity of identified metabolites, multivariate statistical modeling using PCA was performed on mass spectral data. The PCA model showed a variation value (*R* ^*2*^ *X*) of 0.73 and predictive capability (*Q* ^*2*^) of 0.81, indicating the excellence of the PCA model. From the PCA score plots, the metabolite profiles were clearly separated between the three groups (i.e., control, TNF-α-stimulated, and curcumin-treated FLS) (Fig 1A). The principal component (PC) 1 had the largest possible variation (55.6%) between these three groups, explaining the significant separation of metabolite profiles between TNF-α-stimulated group and the control and curcumin-treated group. These results indicated that the metabolite profiles of RA FLS were altered by TNF-α and the altered metabolic pattern of TNF-α-stimulated FLS was recovered to that of control group by curcumin treatment.

A total of 84 metabolites, including fatty acid metabolism derivatives (i.e., myristic acid, oleic acid, 1-monopalmitin, and stearic acid), phosphate, adenosine-5-monophosphate, and malonate positively contributed to PC 1 (Fig 1B). However, 35 metabolites, including amino acids (tyrosine, phenylalanine, and oxoproline), tagatose, threitol, glucose, and galactose negatively contributed to PC1. A total of 63 metabolites, such as linoleic acid, urea, and gluconate positively contributed to PC2. The other 56 metabolites, including myo-inositol, glutamate, aminomalonate, and fumarate negatively contributed to PC2.

**Fig 2:**
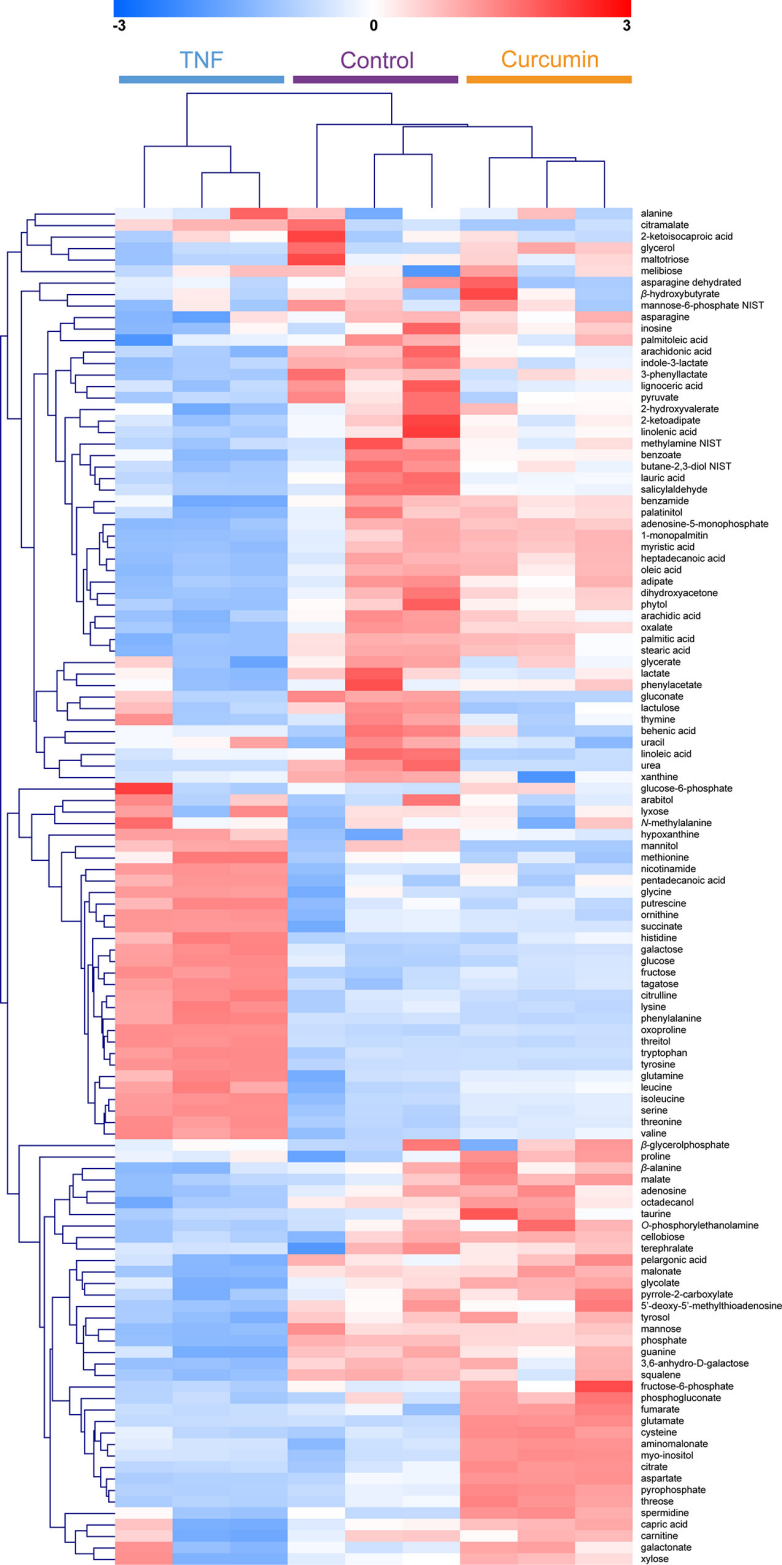
Hierarchical clustering analysis of 119 identified metabolites from RA FLS. The results of heat mapping generated through metabolomic analysis and the relevant changes discovered. A heat map showed that the metabolite profiles of controls were similar to those of the curcumin-treated group. Red color reflects an increase, and blue color a decrease.

A total of 119 identified metabolites were clustered and visualized by the HCA using the Euclidean distance and average linkage method to determine possible variations in the metabolite profiling of the three groups (Fig 2). The clustering of the metabolites led to good separation among the three groups. Similar metabolic patterns were shown between the control and curcumin-treated group. On the other hand, a clear separation of the metabolic profile of the TNF-α-stimulated group from the control and curcumin-treated groups was shown. This indicated that the metabolic pattern of the TNF-α-stimulated group was completely different from those of the control and curcumin-treated groups, similar to the PCA result.

### Significant metabolites for elucidating the effects of TNF-α and curcumin

The metabolomic analysis revealed that many metabolites were severely perturbed when RA FLS were exposed to TNF-α. To compare changes in metabolites between the control group and TNF-α-stimulated FLS, a Student’s *t*-test at the 5% significance level was conducted. The levels of 41 metabolites, including xanthine, palmitic acid, stearic acid, pyruvate, and myristic acid were lower in the TNF-α-stimulated group than those in the control group. Twenty four metabolites, such as oxoproline, tyrosine, threonine, serine, phenylalanine, tryptophan, and valine were higher in the TNF-α-stimulated group than in the control group. Subsequently, the metabolic pattern of the curcumin-treated RA FLS before TNF-α stimulation became similar to that in the control group. Among 65 metabolites altered by TNF-α, the levels of 54 metabolites reached levels similar to those in the control group after curcumin treatment (Table 1).

### Effects of curcumin on TNF-α-induced IL-6, IL-8, MMP-1, and MMP-3 production in RA FLS

**Fig 3:**
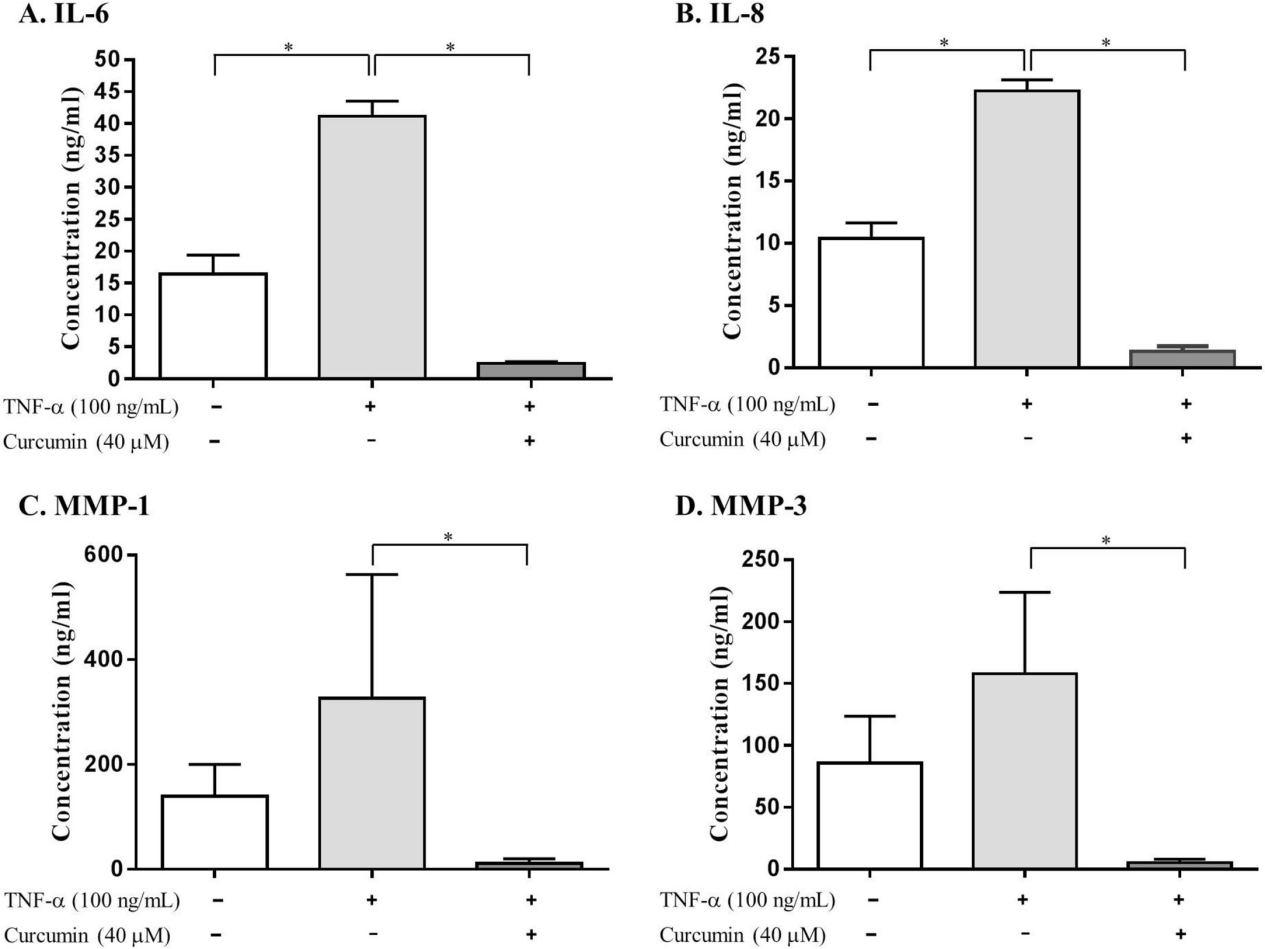
Inhibition of TNF-α-induced production of (A) IL-6, (B) IL-8, (C) MMP-1, and (D) MMP-3 by curcumin in RA FLS. RA FLS (N = 3) were incubated with DMSO-containing vehicle or curcumin for 1 h, followed by stimulation with TNF-α (100 ng/mL) for 24 h. Production of IL-6, IL-8, MMP-1, and MMP-3 in the culture supernatants was measured using a commercially available ELISA kit. Values are mean ± standard deviation. * = *P* < 0.05, by the Kruskal-Wallis test.

Stimulation of RA FLS with TNF-α (100 ng/mL) for 24 h resulted in a 2.5-, 2-, 2.3-, and 1.8-fold induction of IL-6, IL-8, MMP-1, and MMP-3 production, respectively, compared to that in the control group, when measured in culture media using ELISA (Fig 3A–3D). Treatment with curcumin significantly inhibited TNF-α-induced IL-6, IL-8, MMP-1, and MMP-3 production. Interestingly, curcumin resulted in almost 94% inhibition in IL-6 and IL-8 production, and blocked MMP-1 and MMP-3 production by 96%, compared to that in the TNF-α-stimulated FLS.

## Discussion

Our metabolomic analysis using RA FLS reaffirmed that curcumin has a protective effect against the inflammatory response of TNF-α. Exposure to TNF-α caused prominent changes in the intracellular metabolites of RA FLS that was reversed by curcumin treatment. In addition, TNF-α stimulation and curcumin treatment led to perturb metabolite profiling of amino acids and fatty acids. It is suggested that curcumin could weaken obvious metabolic effects of TNF-α exposure in RA FLS and prominent metabolic target of TNF-α and curcumin is amino acid and fatty acid metabolism.

Recently, metabolomic analysis has been used to monitor the activity of tumor cells, the effects of anticancer drugs in tumor cells, and to detect specific genetic alterations in tumor cells [27–29]. RA FLS are key effector cells in the invasion of the synovium and have a role in the initiation and perpetuation of destructive joint inflammation [19]. In this regard, we used the metabolomic approach with GC/TOF-MS to research the role of FLS in RA. This study demonstrated clear differentiation of the metabolic patterns of TNF-α-stimulated FLS from those of the control or curcumin-treated FLS. One of the most important findings is that these differences were identified using an unsupervised analysis, with no information about the samples. Since all three samples were cultured under the same conditions, the observed discrimination demonstrates that the changes in metabolic profile strongly reflect the effects of TNF-α and curcumin. From the PCA plots, TNF-α-stimulated FLS moved from the left to the right after treatment with curcumin, suggesting that metabolic perturbation by TNF-α, which has a role in the pathogenesis of RA, was restored to the metabolic profile of the control group after curcumin treatment. Also, little differences were shown in metabolic profiles between the control and curcumin-pretreated groups by PC2. From the clinical view points, clinicians frequently have experienced that patients with RA does not make a full recovery but have recovered considerably by treating RA with disease modifying drugs. We think that these findings in clinical practice are consistent with making a difference between control group and curcumin-pretreated group by PC2. On the other hand, in Fig 2 and Table 1, there are some metabolites showing significant differences between the control and crucumin-pretreated groups. Further study is required to determine the exact role of each metabolite including anti-inflammatory effects.

**Fig 4:**
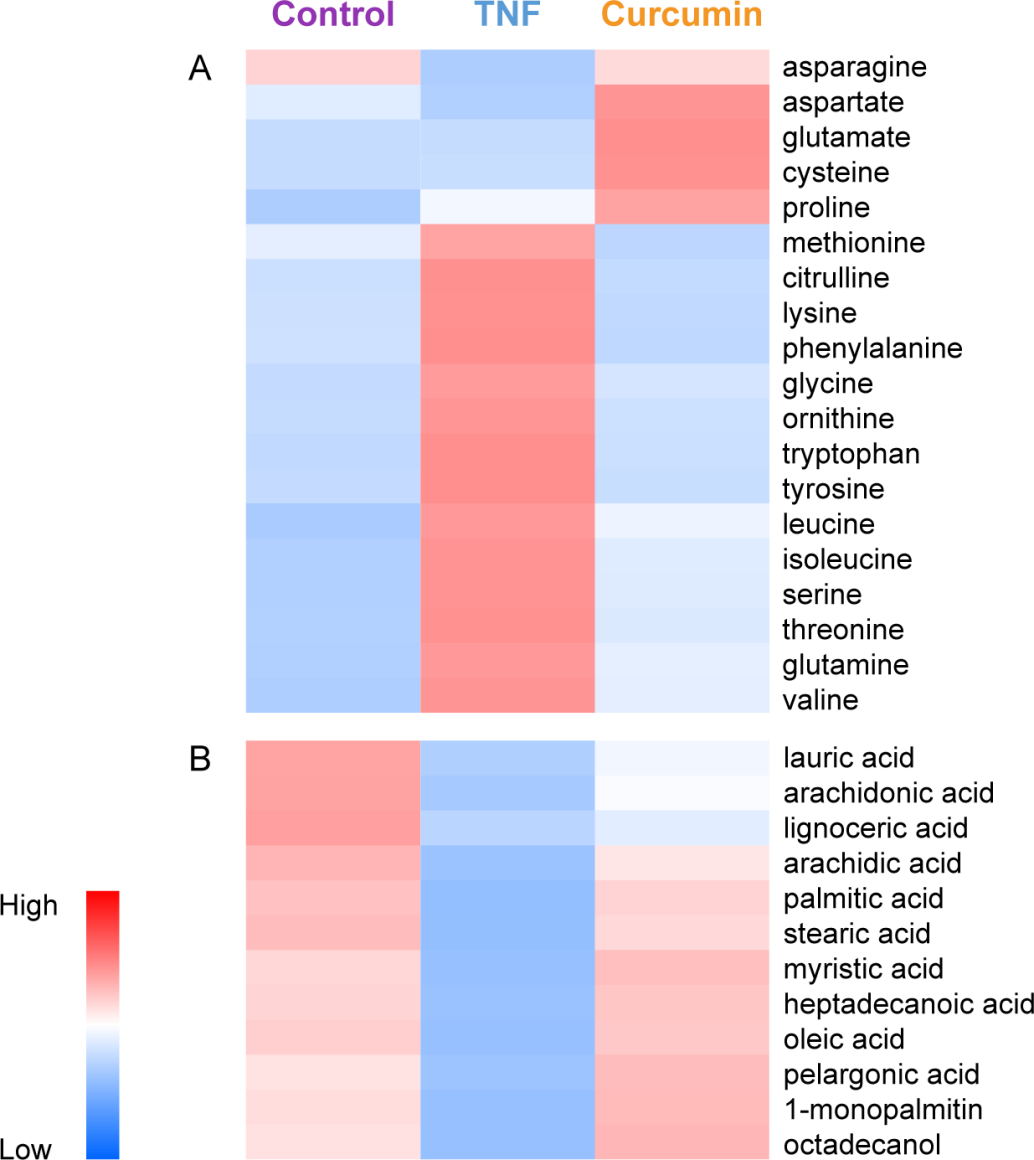
The intensities of metabolites related to amino acid metabolism (A) and fatty acid metabolism (B).

The pathway analysis conducted using MetaboAnalyst based on enrichment analysis and pathway topology analysis to reveal the change of metabolisms by TNF-α and curcumin. These results showed that metabolite levels, which are involved in amino acid biosynthesis, purine metabolism, glycolysis, and fatty acid metabolism, were affected by TNF-α in RA FLS (Table 2), but returned to their original levels after curcumin treatment (Table 3). In our study, most of the glucogenic and ketogenic amino acids levels increased more in the TNF-α-stimulated FLS group than the control group (Fig 4A). The metabolic profiling of RA FLS after TNF-α stimulation was in accordance with a previous study in which RA synovial fluid was used [23]. Treatment with curcumin reversed these TNF-α-induced perturbations of amino acids metabolites to normal. The level of glycine in FLS increased after TNF-α treatment and decreased after curcumin treatment. Glycine has been reported to stimulate prostaglandin E2 and COX-2 in IL-1β-stimulated human gingival fibroblasts [30]. In addition, the levels of citrulline were significantly higher in the TNF-α-stimulated group than in the control group (3.6-fold increase). Similarly, synovial tissue and FLS from RA patients had higher levels of citrullinated protein than those from osteoarthritis patients [31]. Citrulline is an essential constituent of antigenic determinants recognized by RA-specific autoantibodies. Citrullinated proteins may be detected as antigens by the immune system, thereby generating an immune response. http://en.wikipedia.org/wiki/Anti%E2%80%93citrullinated_protein_antibody-cite_note-pmid17558653-3 The high levels of citrulline in TNF-α-stimulated FLS reduced to the level of the control group after curcumin treatment. Collectively, this suggested that the effect of curcumin on preventing joint inflammation may be associated with the restoration of amino acid metabolite levels, such as citrulline or glycine. In addition to the known mechanism of action of curcumin, our metabolomic study revealed the new mechanism of action of curcumin for reducing synovial inflammation.

**Table 2:** The major metabolic pathways changed by TNF-α stimulation compared with the control group.

Pathway	-log (*P*)^a^	Impact^b^
Purine metabolism	12.544	0.075
Histidine metabolism	11.959	0.140
Pyrimidine metabolism	11.868	0.114
Phenylalanine, tyrosine and tryptophan biosynthesis	10.836	0.008
Nitrogen metabolism	10.302	0.008
Ubiquinone and other terpenoid-quinone biosynthesis	10.053	<0.001
Arginine and proline metabolism	9.776	0.359
Sulfur metabolism	9.304	0.000
Phenylalanine metabolism	9.188	0.173
β-Alanine metabolism	9.184	0.334
Aminoacyl-tRNA biosynthesis	9.023	0.169
Glycine, serine and threonine metabolism	8.906	0.421
Methane metabolism	8.858	0.034
Glycolysis or Gluconeogenesis	8.797	0.095
Glyoxylate and dicarboxylate metabolism	8.238	0.172
Glycerolipid metabolism	7.824	0.230
Alanine, aspartate and glutamate metabolism	7.786	0.520
Propanoate metabolism	7.681	0.086
Caffeine metabolism	7.420	0.031
Fatty acid metabolism	7.223	0.030

*P* ^a^ value and Impact^b^ were calculated from enrichment and pathway topology analysis.

**Table 3:** The major metabolic pathways recovered by curcumin-pretreatment compared with TNF-α stimulation group.

Pathway	-log (*P*)^a^	Impact^b^
Valine, leucine and isoleucine biosynthesis	4.213	0.074
Glycerolipid metabolism	4.049	0.230
Aminoacyl-tRNA biosynthesis	4.014	0.169
Nitrogen metabolism	3.974	0.008
Purine metabolism	3.949	0.075
Butanoate metabolism	3.926	0.125
Galactose metabolism	3.915	0.109
β-Alanine metabolism	3.861	0.334
Pantothenate and CoA biosynthesis	3.846	0.073
Propanoate metabolism	3.836	0.086
Glycine, serine and threonine metabolism	3.824	0.421
Cyanoamino acid metabolism	3.819	<0.001
Arginine and proline metabolism	3.799	0.359
Glyoxylate and dicarboxylate metabolism	3.791	0.172
Lysine biosynthesis	3.788	0.159
Fatty acid biosynthesis	3.777	<0.001
Alanine, aspartate and glutamate metabolism	3.763	0.520
Nicotinate and nicotinamide metabolism	3.739	0.038
Citrate cycle (TCA cycle)	3.738	0.185
Phenylalanine metabolism	3.716	0.173

*P* ^a^ value and Impact^b^ were calculated from enrichment and pathway topology analysis.

Accumulated data have revealed that TNF-α perturbs the normal regulation of lipid metabolism [32]. According to our results, total fatty acid levels, including saturated fatty acids (SFAs: arachidic acid, lignoceric acid, lauric acid, myristic acid, palmitic acid, and stearic acid), polyunsaturated fatty acids (PUFAs: arachidonic acid and oleic acid), octadecanol, pelargonic acid, and heptadecanoic acid were significantly lower in the TNF-α-stimulated FLS group than in the control group. On the contrary, the lower levels of SFAs and PUFAs in the TNF-α-stimulated FLS restored to the levels of the control group after curcumin treatment (Fig 4B). SFAs were reported to generate reactive oxygen species (ROS) [33]. Curcumin is a potent in vitro antioxidant owing to its ability to scavenge ROS [34]. These findings suggest that the effect of curcumin as an antioxidant may be affected by the change of SFA abundance. In addition, the levels of arachidonic acid were significantly different between the TNF-α-stimulated and control groups. The levels of arachidonic acid were significantly decreased in the TNF-α-stimulated FLS group compared to that in the control group, while its levels increased in curcumin-treated group. Arachidonic acid mediates inflammation and can be metabolized by COX or lipoxygenase. TNF-α has no apparent effect on the expression of COX-1, but induces the expression of COX-2 [35]. Curcumin inhibited the inflammatory response through the suppression of COX-2 in FLS, followed by the inhibition of prostaglandin E2 synthesis [3,8]. The findings in this study are thought to reaffirm the effect of TNF-α and curcumin on COX-2 with respect to metabolomics. In our study, treatment with curcumin also inhibited TNF-α-induced IL-6, IL-8, MMP-1, and MMP-3 production and restored altered fatty acids levels by TNF-α. Recently, it was reported that free fatty acids, such as palmitic acid, stearic acid, or oleic acid, induce IL-6, IL-8, pro-MMP-1, and MMP-3 in RA FLS [36]. In this context, perturbation of fatty acids levels by TNF-α and curcumin may cause changes in inflammatory cytokines and MMP production. Thus, a reasonable conclusion is that free fatty acids are one of the major components in the mechanism of action of curcumin.

In conclusion, metabolomic data and multivariate statistical analysis clearly revealed strong TNF-α- and curcumin-induced effects on the central metabolism of RA FLS. A prominent metabolic target of TNF-α and curcumin is amino acid and fatty acid metabolism, as revealed by perturbation of metabolite profiles. This is the first report of GC/TOF-MS-based metabolomic application to evaluate the effect of curcumin on FLS which are key effector cells in RA. These results also suggest that metabolomic investigation of FLS has the potential for discovering new biologic targets for therapeutic drugs in RA.

## Supporting Information

S1 File Table A. Demographic and clinical characteristics of patients with rheumatoid arthritis undergoing arthroscopic synovectomy or joint replacement surgery. (DOCX)
