# Supplementary material for: Metabolomic Elucidation of the Effects of Curcumin on Fibroblast-Like Synoviocytes in Rheumatoid Arthritis
Source: PLoS One. 2015 Dec 30;10(12):e0145539. doi: 10.1371/journal.pone.0145539 (PMC4696817; doi:10.1371/journal.pone.0145539)
Supplement: S1 File — (DOCX) [file pone.0145539.s001.docx]

**Supporting Information**

**Table A.** Demographic and clinical characteristics of patients with rheumatoid arthritis undergoing arthroscopic synovectomy or joint replacement surgery

|  | **RA1** | **RA2** | **RA3** |
| --- | --- | --- | --- |
| **Sex** | Female | Female | Female |
| **Age (years)** | 53 | 70 | 38 |
| **DM** | - | + | - |
| **Smoking** | - | - | - |
| **Operation** | Synovectomy | Joint replacement | Synovectomy |
| **Site** | Wrist | Knee | Wrist |
| **BMI** (kg/m^2^) | 26.8 | 23.2 | 25.5 |
| **ESR** (mm/h) | 55 | 25 | 38 |
| **CRP** (mg/dL) | 0.66 | 0.15 | 1.9 |
| **RF** (IU/mL) | 72.7 | 174.6 | 11.3 |
| **ACPA** (U/mL) | 29.3 | N/A | < 7 |
| **DAS28** | 5.22 | 4.92 | 5.24 |
| **Medications** | MTX, SSZ, HCQ, NSAID | MTX, LFN, NSAID | ETN, TAC, SSZ, NSAID, Pd |

Normal range: ESR 0-20 mm/h, CRP < 0.6 mg/dL, RF 0-14 IU/mL, ACPA < 7 U/mL. ACPA, anti-citrullinated protein antibody; BMD, body mass index; ESR, erythrocyte sedimentation rate; CRP, C-reactive protein; DAS28, disease activity score; DM, diabetes mellitus; DMARDs, disease modifying anti-rheumatic drugs; ETN, etanercept; HCQ, hydroxychloroquine; LFN, leflunomide; MTX, methotrexate; NSAID, nonsteroidal anti-inflammatory drug; Pd, prednisolone; RA, rheumatoid arthritis; SSZ, sulfasalazine; TAC, tacrolimus. N/A, not available.
